# The disease burden of pneumoconiosis in China from 1990 to 2021 and projection to 2050 based on GBD 2021: a cross-sectional study

**DOI:** 10.3389/fpubh.2025.1583895

**Published:** 2025-06-25

**Authors:** Xiangwen Gong, Kaiwang Cui, Zhi Liu, Yongtong Tang, Jingbo Li, Youmei Chen, Zhangshun Tu, Miaomiao Yuan, Jianping Liu, Haiwu Wu

**Affiliations:** ^1^Department of Respiratory and Critical Care Medicine, Ganzhou Key Laboratory of Respiratory Diseases, Ganzhou Institute of Respiratory Diseases, The Fifth People’s Hospital of Ganzhou, Ganzhou, China; ^2^Department of Respiratory and Critical Care Medicine, Xingguo County Pulmonary Hospital, Ganzhou, China; ^3^Department of Respiratory and Critical Care Medicine, The People’s Hospital of Shangyou, Ganzhou, China; ^4^School of Rehabilitation Medicine, Gannan Medical University, Ganzhou, China

**Keywords:** pneumoconiosis, global burden of disease, epidemiologic studies, China, cross-sectional study

## Abstract

**Background:**

Pneumoconiosis is a serious occupational disease with high incidence and prevalence in China. This study aimed to describe the long-term trends in the burden of pneumoconiosis and its epidemiological characteristics in China over the past 30 years.

**Methods:**

Data from this cross-sectional study were obtained from the Global Burden of Disease (GBD) 2021 study. Joinpoint was used to calculate the annual average percentage change (AAPC), and age–period–cohort (APC) analyses were used to assess trends in the burden of pneumoconiosis. The Bayesian age-period-cohort (BAPC) model was used to forecast pneumoconiosis burden from 2022 to 2050.

**Results:**

From 1990--2021, the age-standardized incidence rate (ASIR) and mortality rate (ASMR) of pneumoconiosis in China declined from 2.2 (1.83, 2.59) to 1.42 (1.21, 1.63) and from 0.9 (0.73, 1.09) to 0.41 (0.32, 0.53) cases per 100,000 people. The AAPCs of the ASIR and ASMR were −1.42% and −2.15%, respectively. The age-standardized disability-adjusted life years (DALY) and years of life lost (YLL) also decline from 24.32 (20.05, 29.47) to 10.86 (8.49, 13.87) and from 22.39 (18.13, 27.51) to 9.38 (7.1, 12.32) years per 100,000 persons. APC analyses revealed that the incidence and mortality risk of pneumoconiosis increased with age, decreased with period, and were greater in the early birth cohort. From 2022 to 2025, the ASIR and ASPR are expected to remain relatively stable, while the ASMR is projected to decline significantly to 0.267/100,000 in 2050.

**Conclusion:**

Despite the downward trend in age-standardized disease burden indicators of pneumoconiosis, the burden of pneumoconiosis remains heavy due to the large occupational population in China.

## Introduction

Pneumoconiosis is a preventable occupational lung disease caused by the inhalation of dust particles such as coal dust or different types of mineral dust. Its main pathological changes are fibrosis of the lung tissue and chronic lung inflammation (1, 2). The lung damage caused by pneumoconiosis is chronic, progressive, irreversible, and fatal (3, 4). Common types of pneumoconiosis include coal workers’ pneumoconiosis (CWP), silicosis, asbestosis, mixed pneumoconiosis, graphite lung, and talcum lung (5). These conditions are prevalent in high-risk industries such as mining, metal casting, machinery manufacturing, construction, blasting, and quarrying (6). In the early stages of the disease, people with pneumoconiosis are asymptomatic, but as the disease progresses and comorbidities develop, symptoms may emerge.

Pneumoconiosis is widespread worldwide and has become a major health problem that poses a serious threat to global public health (7). The most recent study reported that, despite an overall decline in global pneumoconiosis burden from 1990 to 2021, but pneumoconiosis remains a public health concern (8). China is particularly affected by pneumoconiosis due to its large population and high incidence rates. In 2020, a statistical survey on the current situation of occupational disease hazards in China’s traditional industries revealed that 74.18% of enterprises experienced dust hazards and that 39.36% of workers were exposed to occupational disease hazards (9), indicating that a substantial portion of the workforce is at risk. The Global Burden of Disease study further highlights the heavy burden of pneumoconiosis in China, and according to a previous analysis, more than 60% of new cases of pneumoconiosis occur in China (10). Despite the high incidence of pneumoconiosis over the past few decades, clinical treatments for pneumoconiosis remain limited, seriously affecting the quality of life of patients (11). Moreover, it poses a high risk of disability and premature death (12). This condition has serious impacts on the physical and mental health of patients as well as heavy economic burdens on patients’ families and society (13, 14).

Given the significant disease burden of pneumoconiosis on public health in China, this cross-sectional study specifically focuses on the disease burden of pneumoconiosis in China from 1990 to 2021 and forecasted to 2050, providing a more detailed analysis of the disease burden within the country.

## Methods

### Study data

The GBD has conducted systematic scientific assessments of published, publicly available, and contributed data on the incidence, prevalence, and mortality of diseases and injuries to ensure data reliability and accuracy (15). GBD 2021 provides data on the prevalence and incidence of pneumoconiosis-related diseases and deaths, years of life lost (YLLs), years lived with disability (YLDs), and disability adjusted life years (DALYs) from 1990–2021. In our analysis, pneumoconiosis included silicosis, asbestosis, coal workers’ pneumoconiosis, and other forms of pneumoconiosis. When we divided the age groups, we divided them into age groups every 5 years. Ethical approval or informed consent was not required for this study, as we used publicly available databases.

### Joinpoint regression model

A joinpoint regression model is a statistical method for analyzing the change in trend of a time series by segmenting the data into multiple linear segments, identifying the connecting points where the trend changes significantly, and locating the point in time where the trend changes significantly, which has been described in detail previously (16). The number of connected points during model fitting was determined automatically by the permutation test or information criterion. The slopes of the segments are fitted using the least squares method. The annual percentage change (APC), average annual percentage change (AAPC) and 95% confidence interval (CI) were calculated. The APC and AAPC were significant if the 95% CI did not include zero (17). The analysis was conducted via the Joinpoint Regression Program version 5.1.0. This software is a tool developed by the Surveillance Research Program of the U.S. National Cancer Institute and is specifically designed for the detection of trends and the identification of significant changes in the trajectory of data over time. It employs a permutation-based method to assess the presence of joinpoints, which are points at which the trend changes direction or rate.

### Age-period-cohort model

Age-Period-Cohort (APC) models are based on the systematic decomposition of population health indicators over age, time and birth cohort, and use a statistical regression framework to distinguish the independent effects of the three, which has been described in detail previously (18). APC models quantify the independent contribution of each effect by constructing a log-linear model or a generalized linear model, and by incorporating constraints to solve the problem of linear dependence of the three. The model results can reveal the drivers of disease risk and are commonly used in epidemiologic and public health research (19). Thus, we used APC modeling to estimate the independent effects of age, period, and birth cohort on the burden of pneumoconiosis disease.

### Bayesian age-period-cohort model

The Bayesian age-period-cohort (BAPC) model is a time-trend analysis method based on the Bayesian statistical framework for decomposing and predicting the incidence of a disease or the incidence of an event. The BAPC model has been widely validated and applied in epidemiological studies, and is capable of dynamically integrating the age, period, and cohort effects to generate predictions with confidence intervals that quantify uncertainty (20). Thus, we used BAPC model to project further disease burden of pneumoconiosis from 2022 to 2050 year.

### Statistical analysis

This study descriptively analyzed the trends in the disease burden of pneumoconiosis in China from 1990 to 2025, using Joinpoint regression model, APC model and BAPC model. Statistical analysis and visualization of the data in this study were performed via R (version 4.3.2) and Joinpoint (version 5.1.0). A *p* value <0.05 was considered to indicate statistical significance.

## Results

### Descriptive analysis

In China, the number of new cases, patients, and deaths from pneumoconiosis rose from 19,441, 114,634 and 7,079, respectively, in 1990 to 29,701, 214,719 and 8,214, respectively, in 2021. The DALYs, YLDs, and YLLs were 221,320, 223,638, and 204,261, respectively, in 2021 and increased to 17,059, 31,644, and 191,993, respectively, in 2021.

However, from 1990 to 2021, all age-standardized indicators of pneumoconiosis in China have shown a decreasing trend, with the age-standardized prevalence rate (ASPR) decreasing from 13.07/100000 to 10.07/100000, the age-standardized incidence rate (ASIR) from 2.2/100,000 to 1.42/100,000, the age-standardized mortality rate (ASMR) from 0.9/100,000 to 0.41/100,000, the DALY from 24.32/100,000 to 10.86/100000, the YLD from 1.93/100,000 to 1.48/100,000, and the YLL from 22.39/100,000 to 9.38/100,000 (Table 1).

**Table 1:** All-age cases, age-standardized prevalence, incidence, mortality, YLLs, YLDs, and DALYs rates of pneumoconiosis in China, 1990–2021.

Measure	1990 All-ages cases	1990 Age-standardized rates per 100,000 people	2021 All-ages cases	2021 Age-standardized rates per 100,000 people
Incidence	19,441 (16,111, 23,023)	2.20 (1.83, 2.59)	29,701 (25,358, 34,290)	1.42 (1.21, 1.63)
Prevalence	114,634 (90,298, 142,740)	13.07 (10.31, 16.25)	214,719 (172,314, 263,366)	10.07 (8.17, 12.31)
Deaths	7,079 (5,747, 8,685)	0.90 (0.73, 1.09)	8,214 (6,296, 10,725)	0.41 (0.32, 0.53)
DALYs	221,320 (180,837, 267,617)	24.32 (20.05, 29.47)	223,638 (174,230, 286,782)	10.86 (8.49, 13.87)
YLDs	17,059 (11,245, 24,709)	1.93 (1.28, 2.76)	31,644 (20,963, 44,760)	1.48 (0.98, 2.10)
YLLs	204,261 (164,956, 251,082)	22.39 (18.13, 27.51)	191,993 (144,666, 254,593)	9.38 (7.10, 12.32)

DALYs, disability-adjusted life-years; YLDs, years lived with disability; YLLs, years of life lost.

**Figure 1:**
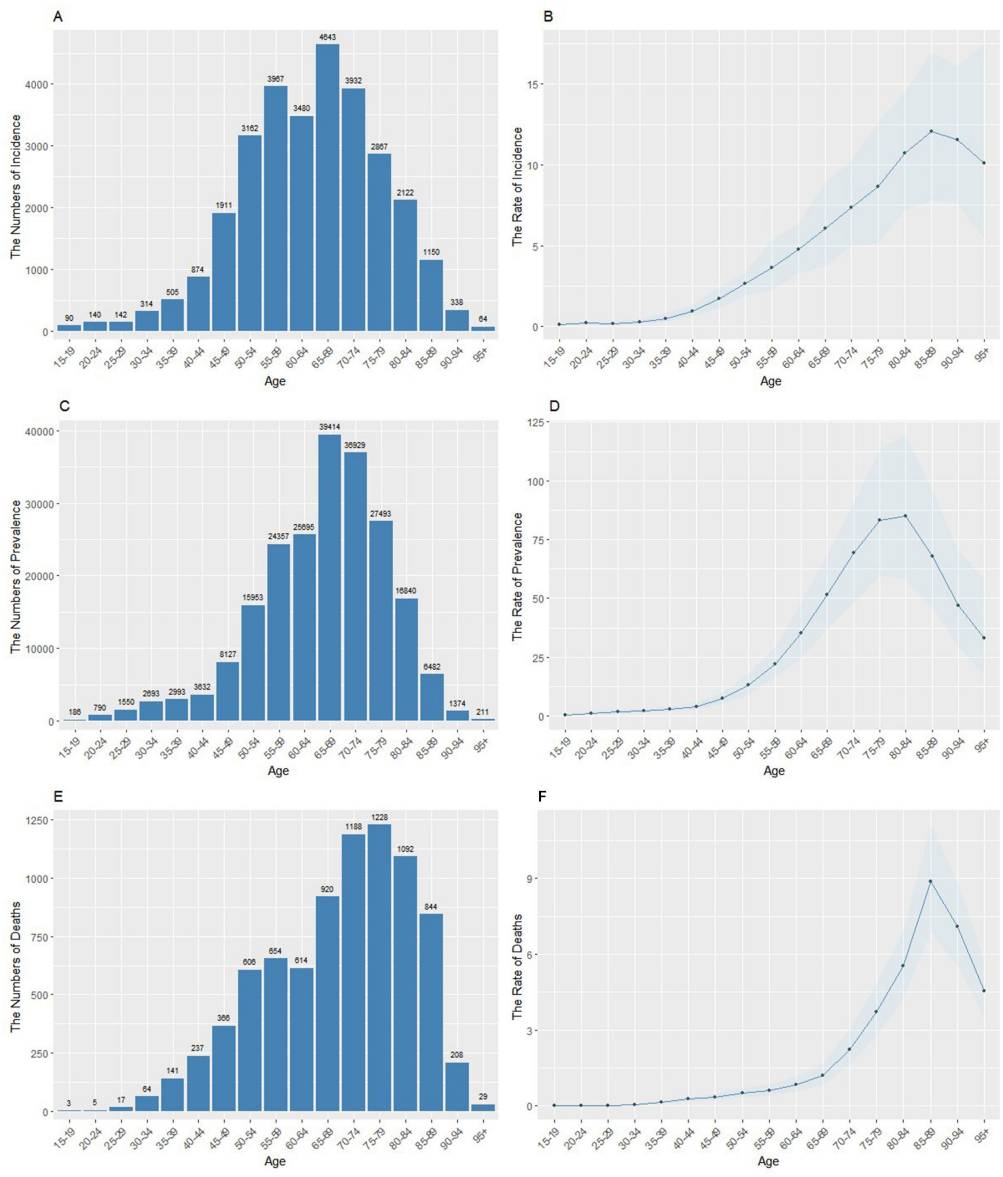
The age-specific number and rates of incidence, prevalence, and deaths of pneumoconiosis in China in 2021. **(A)** Age-specific incidence number. **(B)** Age-standardized incidence rate. **(C)** Age-specific prevalence number. **(D)** Age-standardized prevalence rate. **(E)** Age-specific mortality number. **(F)** Age-standardized mortality rate.

Figure 1 shows the number of new pneumoconiosis cases, patients and deaths in different age groups across China in 2021. The incidence and number of deaths increased quickly after the age of 50 years, with the highest incidence peaks occurring between the ages of 65 and 69 years, whereas the number of deaths increased faster after the age of 65 years and peaked at 75--79 years. Similar tendencies were observed in the rates of incidence, prevalence, and mortality. However, the peak ages were relatively late, specifically 85--89 years, 80--84 years, and 85--89 years.

### Joinpoint regression analysis

**Figure 2:**
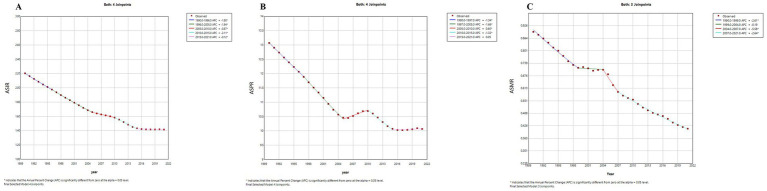
The age-standardized incidence and mortality rates of pneumoconiosis in China from 1990 to 2021. **(A)** Age-standardized incidence rate; **(B)** Age-standardized prevalence rate; **(C)** Age-standardized mortality rate.

According to the results of joinpoint regression analysis, the ASIR, ASPR and ASMR of pneumoconiosis in China generally decreased from 1990--2021 (Figure 2). The incidence trend declined rapidly from 1990--2015, whereas the downward trend weakened from 2015--2021 (APC = −0.12, *p* < 0.05). Although the ASPR decreased most of the time, it increased slightly from 2005--2010 (APC = 0.60, *p* < 0.05) and 2015--2021 (APC = 0.05, *p* < 0.05). The ASMR decreased steadily overall but sharply decreased from 2004--2007 (APC = −5.60, *p* < 0.05) and slightly decreased from 1999--2004 (APC = −0.19, *p* < 0.05).

The AAPCs of incidence, incidence, and mortality rates for pneumoconiosis in China were −1.42 (−1.44–−1.40), −0.84 (−0.87, −0.81), and −2.53 (−2.71, −2.35), respectively, from 1990--2021 (Table 2).

**Table 2:** Joinpoint regression analysis: Trends in age-standardized incidence, prevalence and mortality rates (per 100,000 population) in China, 1990–2021.

ASIR			ASPR			ASMR		
Period	APC (95%CI)	AAPC (95%CI)	Period	APC (95%CI)	AAPC (95%CI)	Period	APC (95%CI)	AAPC (95%CI)
1990–1996	−1.80 (1.85–−1.76)	−1.42 (−1.44—-1.40)	1990–1997	−1.34 (−1.38, −1.29)	−0.84 (−0.87, −0.81)	1990–1999	−2.61 (−2.80, −2.41)	−2.53 (−2.71, −2.35)
1996–2005	−1.94 (−1.97. 1.91)		1997–2005	−1.66 (−1.70, −1.61)		1999–2004	−0.19 (−0.72, 0.35)	
2005–2010	−0.87 (−0.95–-0.79)		2005–2010	0.60 (0.49, 0.70)		2004–2007	−5.58 (−7.09, −4.04)	
2010–2015	−2.11 (−2.18–−2.03)		2010–2015	−1.32 (−1.42, −1.22)		2007–2021	−2.64 (−2.75, −2.53)	
2015–2021	−0.12 (−0.16–−0.08)		2015–2021	0.05 (−0.00, 0.10)				

### Age-period-cohort analysis

**Figure 3:**
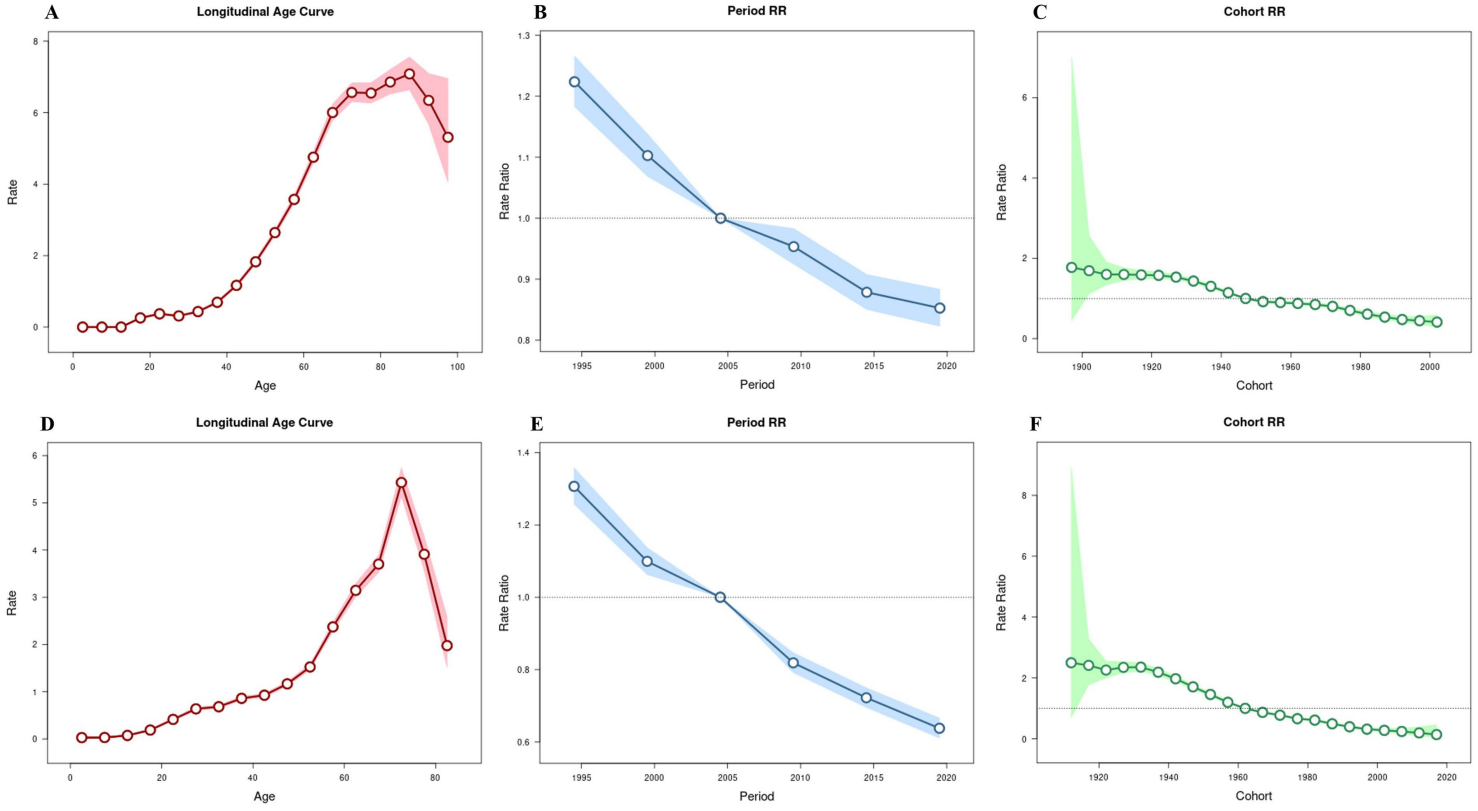
Age-period-cohort analyses of pneumoconiosis incidence and mortality in China. **(A)** Age effect of incidence. **(B)** Period effect of incidence. **(C)** Cohort effect of incidence. **(D)** Age effect of mortality. **(E)** Period effect of mortality. **(F)** Cohort effect of mortality.

The effects of age, period, and birth cohort on pneumoconiosis incidence are shown in Figure 3. In terms of the effects of age on pneumoconiosis morbidity and mortality in China, both morbidity and mortality increase significantly with age. Morbidity peaked in the 85–90-year-old group, with a risk ratio (RR) of 7.88 (95% CI 7.37–8.43). The effect of age on mortality showed a similar trend, with a significant increase in mortality in the 60 years and older groups, with the highest RR of 5.43 (95% CI 5.14--5.74) occurring at 85--90 years.

The effect of period on morbidity and mortality decreased at a constant rate over time, without significant fluctuations. Compared with those in 2002--2006, the RRs of morbidity and mortality were 1.22 (95% CI 1.18--1.27) and 1.31 (95% CI 1.26--1.36) in 1992–1996, respectively. In contrast, the RRs of morbidity and mortality were 0.85 (95% CI 0.82–0.88) and 0.64 (95% CI 0.61–0.67) in 2017–2021, respectively.

Cohort effects on incidence and death showed a continuous downward trend in risk from the early birth cohort to the later birth cohort. The RR of incidence decreased more slowly than that of death.

### Bayesian age-period-cohort model

**Figure 4:**
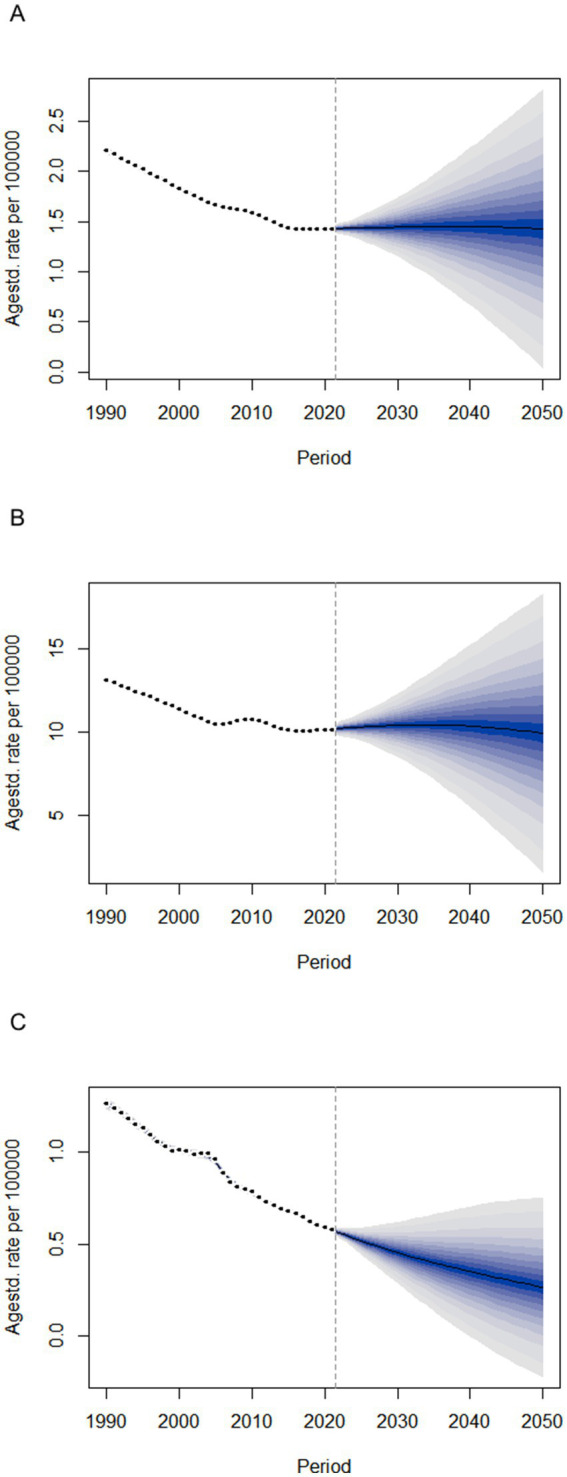
The trends of disease burden of pneumoconiosis in China from 2022 to 2050 predicted by Bayesian age-period-cohort model. **(A)** Age-standardized incidence rate; **(B)** Age-standardized prevalence rate; **(C)** Age-standardized mortality rate.

Figure 4 projects the future disease burden of pneumoconiosis in China up to 2050 using a Bayesian age-period-cohort model. From 2022 to 2025, the ASIR and ASPR are expected to remain relatively stable, at approximately 1.44/100,000 and 10.28/100,000, respectively. However, the ASMR is projected to decline significantly from 0.561/100,000 to 0.267/100,000, with an annual decrease of −2.62%.

## Discussion

This cross-sectional study found that (1) From 1990 to 2021, the number of pneumoconiosis cases, patients, and deaths in China increased, while the age-standardized indicators all decreased. (2) Joinpoint regression analysis showed fluctuating downward trends in these rates, with varying rates of change across different periods. (3) Age-period-cohort analysis revealed that morbidity and mortality increase significantly with age. The effect of period on morbidity and mortality decreased steadily over time. Cohort effects showed a continuous downward trend in risk from earlier to later birth cohorts. (4) Projecting from 2022 to 2025, ASIR and ASPR are expected to remain stable, while ASMR is anticipated to decline significantly. These findings are significant for understanding the progression of pneumoconiosis, assessing the effectiveness of past and current prevention strategies, and guiding the development of future policies and interventions to further reduce the burden of this disease.

Our study revealed that while the absolute burden of pneumoconiosis increased from 1990--2021, the age-standardized rates of incidence, prevalence, mortality, and DALY declined in general. This indicates that some progress has been made in the control and management of pneumoconiosis in China, especially in reducing the mortality rate (21, 22). However, the rate of decline in disease burden in recent years has slowed, particularly AISR, and the APC of ASPR increased from 2015--2021, suggesting that further strengthening of protective and preventive strategies is necessary to sustain and accelerate this downward trend.

There are marked differences in the burden of pneumoconiosis among different age groups in China, especially among middle-aged and older adult individuals. This may be because middle-aged and older adults may have spent long periods in high-risk jobs, such as mining and construction, during their youth but only develop noticeable symptoms after middle age, and this effect becomes more pronounced as they age (23). These data highlight the cumulative effects of exposure to high-risk occupations and long-term occupational exposure on pneumoconiosis (24). Moreover, the lack of proper protection measures in the past may have contributed to the higher incidence of pneumoconiosis among middle-aged and older adult workers.

Over the past 30 years, China’s rapid urbanization process has outpaced the development of robust occupational health and safety measures, leading to enormous challenges in terms of occupational diseases (25). The large Chinese occupational population and the relative lag in pneumoconiosis control strategies compound these challenges (26). Relevant units should take action according to the relevant regulations to address the dust hazards generated during the construction process properly, starting from the root causes (27). Previous studies have shown that workers’ compensation insurance fails to provide coverage for most pneumoconiosis miners (28). Policymakers need to formulate and improve relevant laws and regulations, establish a sound healthcare system, clarify the responsibilities of enterprises, and ensure that they strictly implement dust control measures. Moreover, early screening and comprehensive disease assessment of pneumoconiosis are key aspects of prevention and control, and there is a need to strengthen occupational health monitoring and early intervention for workers in high-risk occupations to detect pneumoconiosis at an early stage.

Fibrosis of lung tissue is irreversible and irreversible, with many complications and a long treatment cycle, coupled with the fact that pneumoconiosis is a progressive disease, a chronic disease requiring lifelong symptomatic and rehabilitative treatment, and that people of lower socioeconomic status will bear a greater burden of the disease (29, 30). Therefore, the prevention and control of its complications in clinical practice is crucial for the prognosis of patients with pneumoconiosis (31). Clinical symptoms of pneumoconiosis can be relieved, and quality of life may be improved by several treatments (32). Under current standards of care, the majority of patients do not have access to adequate or timely treatment and rehabilitation options, and there is a lack of access to occupational health services and medical services (33). Pulmonary rehabilitation improves patients’ exercise capacity, dyspnea severity, and health-related quality of life (34). However, its utilization is low, and in rural areas, access to pulmonary rehabilitation programs may not be available (35). Active utilization of rehabilitation resources, through the use of appropriate rehabilitation interventions, will help improve the quality of life of patients and enable them to better return to society to reduce the burden of pneumoconiosis disease (21). In addition to the prevention and treatment of pneumoconiosis, researchers need to further elucidate the mechanisms underlying pneumoconiosis and identify new biomarkers to assess the severity of the patient’s condition more accurately and predict the development of the disease more accurately.

### Limitations

There are inevitably several limitations to this study. First, all the information is based on the GBD 2021 database; therefore, accuracy depends on the quality of the data. Second, owing to the varying diagnostic criteria and classifications of pneumoconiosis over time, there may be statistical errors in the data, and it is possible that some patients have not been diagnosed and reported. Third, the lack of detailed data at the provincial level from the GBD database prevented further assessment of disease burden differences between provinces. Despite these limitations, our study provides a reliable basis for the management of pneumoconiosis.

## Conclusion

In summary, although the age-standardized disease burden indicators of pneumoconiosis in China were declined from 1990 to 2021, the increasing number of pneumoconiosis patients and the projected stable ASIR and ASPR indicate the burden of pneumoconiosis remains a persistent challenge. It therefore highlights the need for sustained preventive and control measures, especially for occupational workers involved in dusty environments, to reduce the future burden of pneumoconiosis.

## Data Availability

The original contributions presented in the study are included in the article/supplementary material, further inquiries can be directed to the corresponding author.
